# *In silico* analysis of hypoxia activated prodrugs in combination with anti angiogenic therapy through nanocell delivery

**DOI:** 10.1371/journal.pcbi.1007926

**Published:** 2020-05-28

**Authors:** Cameron Meaney, Sander Rhebergen, Mohammad Kohandel

**Affiliations:** Department of Applied Mathematics, University of Waterloo, Waterloo, Ontario, Canada; University of California Irvine, UNITED STATES

## Abstract

Tumour hypoxia is a well-studied phenomenon with implications in cancer progression, treatment resistance, and patient survival. While a clear adverse prognosticator, hypoxia is also a theoretically ideal target for guided drug delivery. This idea has lead to the development of hypoxia-activated prodrugs (HAPs): a class of chemotherapeutics which remain inactive in the body until metabolized within hypoxic regions. In theory, these drugs have the potential for increased tumour selectivity and have therefore been the focus of numerous preclinical studies. Unfortunately, HAPs have had mixed results in clinical trials, necessitating further study in order to harness their therapeutic potential. One possible avenue for the improvement of HAPs is through the selective application of anti angiogenic agents (AAs) to improve drug delivery. Such techniques have been used in combination with other conventional chemotherapeutics to great effect in many studies. A further benefit is theoretically achieved through nanocell administration of the combination, though this idea has not been the subject of any experimental or mathematical studies to date. In the following, a mathematical model is outlined and used to compare the predicted efficacies of separate vs. nanocell administration for AAs and HAPs in tumours. The model is experimentally motivated, both in mathematical form and parameter values. Preliminary results of the model are highlighted throughout which qualitatively agree with existing experimental evidence. The novel prediction of our model is an improvement in the efficacy of AA/HAP combination therapies when administered through nanocells as opposed to separately. While this study specifically models treatment on glioblastoma, similar analyses could be performed for other vascularized tumours, making the results potentially applicable to a range of tumour types.

## Author summary

Tumour hypoxia is a well-documented phenomenon with adverse effects for the progression of the cancer. Accordingly, various therapeutic strategies have emerged in recent years to combat its effects. Herein, we present an experimentally-motivated mathematical model used to assess the feasibility of the therapeutic combination of anti angiogenic agents with hypoxia-activated prodrugs. Analysis of the combination therapy shows that delivery through drug nanocells provides the optimal anticancer effect: a novel result which should inspire further examination.

## Introduction

Hypoxia is a common feature of solid tumours resulting from an inadequate oxygen supply and has been associated with many negative cancer behaviours including increased metastasis and aggressive phenotypes, promotion of genetic instability, and decreased treatment effectiveness for immunotherapy, radiotherapy, and chemotherapy [1–11]. Accordingly, strategies to combat tumour hypoxia are in high demand. On the other hand, tumour hypoxia has gained significant interest in recent years for its potential as a target for selective drug delivery in cancer.

### Hypoxia-activated prodrugs

Hypoxia-activated prodrugs (HAPs) have emerged as a method for selective targeting of tumours through the exploitation of their hypoxic cores. HAPs are bioreductive compounds which remain inactive under normoxic conditions, but are metabolized under hypoxic conditions within the body into their cytotoxic forms. Their hypoxic selectivity is achieved through a 1e^−^ or 2e^−^ reduction reaction which is rapidly reversed under an abundance of oxygen, but serves as the first step in a reduction cascade under hypoxia. Importantly, this activation exclusively in hypoxic zones does not prevent HAPs from attacking non-hypoxic tumour cells. Once activated in hypoxic regions, HAPs are able to diffuse back into non-hypoxic regions and attack normoxic tumour cells—a phenomenon termed the Bystander Effect (although there is some debate regarding the overall importance of these bystander effects [12–14]). In the present study, we focus on the nitroimidazole-based HAP, TH-302 (Evofosfamide), which undergoes a 1e^−^ reduction to form the DNA cross-linking bromo-isophosphoramide mustard (Br-IPM) under hypoxia [15, 16].

Numerous experimental preclinical studies have analyzed the use of HAPs alone or in combination with other therapies, showing positive outcomes for the control of tumour growth and invasion in a number of different tumour types ([12, 13, 17–22] for example). Furthermore, mathematical models of HAP action have been developed which are able to accurately reproduce experimental results ([23–27] for example). Despite the theoretical understanding and promising traits of HAPs, clinical success has been hard to achieve as many HAPs have failed recent clinical trials [15, 16, 28]. In the case of TH-302, clinical trials have been ongoing for both its use in monotherapy and combination therapy for many years. TH-302 monotherapies have generally showed limited results in phase I/II clinical trials, whereas its combination with radiation and/or other chemotherapeutics has had more positive results with the outcomes of ongoing trials eagerly awaited (see [29, 30] for recent overviews). Fortunately, there is genuine optimism in the field with the development and improvement of technologies such as medical imaging, biomarking, and genetic screening [16, 28]. Moreover, the disconnect between theory and practice is thought to be mainly a symptom of inappropriate patient selection, as treatment efficacy will be based on patient levels of hypoxia and nitroreductase expression [28, 31]. Nonetheless, HAPs also suffer from many of the same problems as conventional therapeutics. Most notable of these is the inefficient delivery of drugs to the tumour core due to insufficient blood supply and high interstitial fluid pressures caused by dysfunctional tumour vasculature.

### Anti angiogenic agents

**Fig 1:**
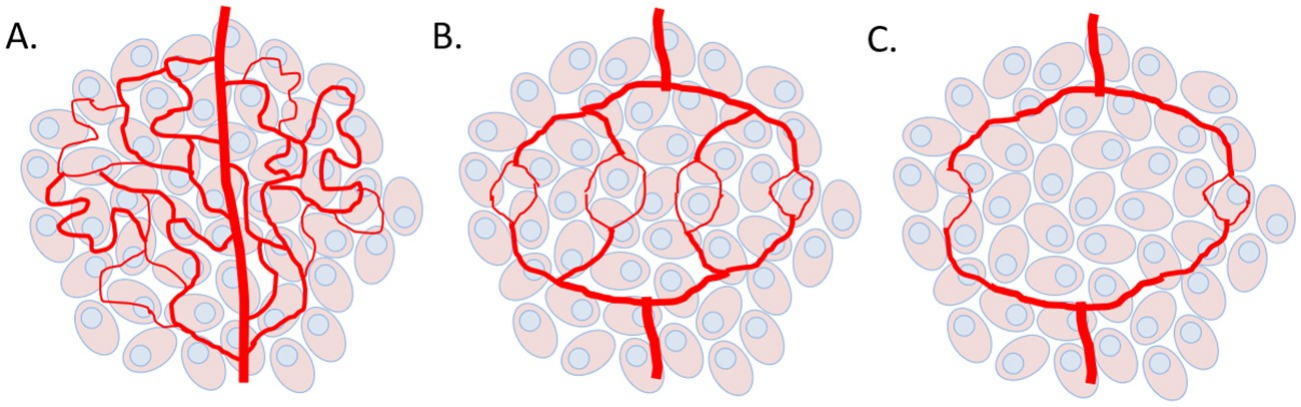
Cases of tumour vascularization. In A. the tumour is overvascularized with chaotic, irregular vessels (*m* > 1) causing perfusion-limited hypoxia. In B. vascular normalization (*m* ≈ 1) is achieved where optimal oxygen or drug extravasation is realized. In C. a strong dose of AAs causes vascular destruction (*m* < 1), leading to diffusion-limited hypoxia in the tumour. The quantity *m* is the tumour blood vessel density.

Tumour vasculature is abnormal in a number of ways, including differences in the general structure and the specific behaviours of cells. Tumours achieve these differences largely through an up-regulation of pro-angiogenic factors such as vascular endothelial growth factor (VEGF) and tumour growth factor-*β* (TGF-*β*) and a down-regulation of anti angiogenic factors such as Thrombospondin-1 (TSP-1), causing the ‘angiogenic switch’ to be permanently switched to ‘on’ [32–35]. Tumour vessels branch irregularly, forming a chaotic web of interconnecting paths which impede blood flow. Furthermore, the endothelial cells lining the vessel walls of tumours are often able to detach or stack on one another, leading to openings in the vessel wall. These openings not only enhance the ability of tumour cells to enter the blood stream, but contribute to the leakiness of vessels, allowing blood to pool in tissues and increase fluid pressures in the interstitium. These pressures prevent blood from exiting at the proper spots and delivering drugs or essential nutrients such as oxygen. See a graphic representation of the cases of tumour vascularization in Fig 1.

Notably, the inefficient delivery from tumour vessels is often not due to a reduction in the blood volume flowing through the tumour system (the number of vessels in the area). Instead, the main culprit is the inability of molecules to be properly delivered from vessels to tumour tissue due to the previously mentioned vascular irregularities. In the case of oxygen, hypoxic zones may be caused by two separate mechanisms. Some hypoxic regions may simply be a result of a lack of nearby blood vessels—which we refer to as diffusion-limited hypoxia—however, other regions become hypoxic due to inefficient delivery from a nearby vessel—which we refer to as perfusion-limited hypoxia. This has lead to the idea of tumours being ‘over-vascularized’, as the up-regulation of pro-angiogenic factors causes unregulated angiogenesis. From this has stemmed the notion of vessel normalization: the strategy of using anti angiogenic agents (AAs) to prune (rather than destroy) the tumour vasculature in attempt to improve the delivery of cytotoxic drugs to the tumour area. This normalized state appears for only a small amount of time after which the over-vascularization reappears, again causing decreased oxygen and drug delivery. This, in turn, activates Hypoxia-Inducible Factor (HIF-1*α*), which causes an up-regulation of pro-angiogenic factors. This has been termed the ‘normalization window’ and has been the subject of experimental ([36–38]) and mathematical ([39, 40]) studies. Together, these works have highlighted the importance of precise timing and dosing in administrations of combination therapies. Importantly, anti angiogenic vessel normalization strategies have been tested in many different tumour types including brain, breast, colorectal, gastric, lung, and others, making mathematical analyses of their action widely applicable [41].

### Combination therapies

There are few experimental studies examining AA/HAP combinations, and these are compared with our model predictions in the discussion below. The strategy generally employed by these studies is to give a high dose of AAs in order to increase the level of hypoxia and therefore HAP activation. Although this may seem beneficial for the application of HAPs, the elimination of the tumour vessels prevents proper delivery of the HAPs from vessel to tumour. It was for this reason that using AAs for vessel normalization was initially suggested. However, the incorporation of normalization creates a paradox of optimal sequencing. In particular, if AAs are first applied to prune the vasculature and increase HAP delivery, then the drug activation will be hindered through the simultaneous increase in oxygen delivery (due to normalization of the vasculature). Conversely, if HAPs are applied first, then the aforementioned inefficient delivery will remain a hindrance. Similarly, the simultaneous administration does not avoid the trade-off between delivery and activation as if the HAPs do arrive during the normalization window, then they will arrive during optimal oxygen delivery as well.

### Drug nanocells

To solve this dilemma, the use of redox-responsive nanocarriers is examined as a delivery vehicle for combination therapies involving AAs and HAPs. These nanocarriers were first examined by Sengupta *et al* [42] who published a work detailing delivery via a ‘nanocell’ for the chemotherapeutic agent doxorubicin with the AA combretastatin. The nanocell, as described by [42], is a phospholipid envelope coating a nanoparticle which is reversibly bound to a chemotherapeutic agent. An AA lies within the phospholipid envelope and is unbound to the nanoparticle. Deterioration of the envelope results in an initial release of the AA affecting the tumour vasculature followed by the subsequent release of the chemotherapeutic. This method of administration allows for precise control over the temporal release of the drugs which was shown to increase the treatment efficacy from the separate administration case. The initial release of the AA causes a collapse of the tumour vasculature, leaving the subsequently released cytotoxin trapped within the tumour tissue. In [42], it is also suggested that nanocell administration technology could be extended to include additional chemotherapeutic agents, but experimental validation is only done for doxorubicin. In this paper we focus on HAPs and note that to the best of our knowledge, there are no experimental studies comparing separate and nanocell delivery for HAPs to tumours. Theoretically, the nanocell administration of HAPs can be effective as they allow for an escape from the previously mentioned paradox by allowing HAPs to take advantage of both improved delivery and high activation rates. This is done through the delayed release of the HAP after the AA. The action of the AA causes a normalization of the tumour vasculature, allowing the remaining nanoparticle to be delivered from the vessels to the tumour tissue unimpaired. Then, the release of the HAP from the nanoparticle is delayed until the re-collapse of the vasculature and the subsequent re-emergence of hypoxia. Once the HAP has then been released from the nanoparticle, it is trapped within a hypoxic region where it will benefit from high activation. Theoretically, the use of nanocells can give the AA/HAP combination high delivery and activation, but clearly precise timing and dosing is crucial to its effectiveness.

### Paper outline

Several preclinical studies have suggested that currently available angiogenesis inhibitors are unlikely to yield significant sustained improvements in tumor control on their own, but rather will need to be used in combination with other treatments to achieve maximal benefit [43–46]. As the use of HAPs increases, experimental and clinical explorations into their combination with AAs will inevitably appear. We therefore think it timely for applications of their combination to be examined to inform future studies and the feasibility of eventual translation to the clinic. We present here a mathematical model of AA/HAP combination treatments both in separate and nanocell delivery. We focus on the HAP, TH-302, and the AA, combretastatin, for the purposes of model form and parameter values. The proposed treatment is modelled on a malignant glioblastoma included in the mathematical model through equation forms and parameter values (originally estimated from [38] who ran experiments using a mouse xenograft model derived from a U87 MG cell line).

In the Materials and Methods section, our mathematical model is explained for both the separate and nanocell administration cases. Included here is some discussion on choices for model form and parameter values. Following this, the Results section gives the results of our model simulations. First, we provide examples of our model reproducing known results in order to establish agreement with previous literature on the subject. Then, the novel results of our model are given, specifically showing that nanocell administration provides an increased efficacy over the separate administration case. In the Discussion section, the novel results of our model are put into context, including relating them to previous works and discussing limitations and possible extensions. Finally, the contents of the paper are summarized in the Conclusion section.

## Materials and methods

The mathematical model presented here is a combination of existing mathematical models, used here to investigate a novel problem; namely, AA/HAP combinations. It incorporates results from previously published experimental data, informing selections for equation forms and parameter values. It consists of a system of reaction-diffusion equations for the essential components of the tumour growth and treatment system. The parameter meanings, values, and sources for separate administration can be found in Table 1 and for nanocell administration in Table 2. Most of the parameters are taken directly from previous mathematical modelling studies which estimated the parameters from experimental data. Parameters describing tumour cell growth, vasculature development, and oxygen distribution were taken from Kohandel *et al* 2007 [47] who estimated the parameters based off of the experimental results of Winkler *et al* 2004 [38] who examined mouse xenograft models from the U87 MG cell line (glioblastoma). This experimental work showed that selective VEGF blockade can induce a normalization window during which chemotherapy and radiotherapy achieves the best outcome. For parameters describing the distribution and effect of the AA combretastatin, we rely on the reported values from Yonucu *et al* 2017 [40] who matched parameters from the experimental results of [48, 49] and estimated some themselves. Parameters describing the HAP TH-302 were taken from Meaney *et al* 2019 [27] who fitted the parameters by matching to post-treatment images obtained through immunofluorescence staining techniques on a H460 xenograft tumour. Radiation parameters were taken from Powathil *et al* 2012 [50] and nanocell parameters were taken from Kohandel *et al* 2011 [39]. Since mathematical terms for cellular oxygen consumption and nanocell decay were not included in previous modelling studies, the values of the relevant rates were estimated by attempting to match with existing experimental results (as explained in Table 1). To study the effect of parameter uncertainty on our model results, a sensitivity analysis is included in S1 Text, S1 and S2 Figs. Parameters describing treatment are chosen to match previous results in the works from which they come, and a similar sensitivity analysis is performed. These sensitivity analyses show the model to be robust with respect to changes in the parameter values (see details of sensitivity analysis in S1 Text, S1 and S2 Figs). The model is first shown for the case of separate administration of combretastatin and TH-302 (Eqs (1)–(7)), then for the nanocell administration (Eqs (8)–(10)).

**Table 1:** Model parameters.

Parameter	Symbol	Value (Unit)	Reference
Cell Growth			
Diffusivity of Tumour Cells	*D*_*n*_	3.50e-2 (mm^2^/day)	[47]
Cell Proliferation Rate	*r*	0.35 (1/day)	[47]
Cell Carrying Capacity	*n*_*lim*_	2.00e6 (1/mm^2^)	[47]
Proliferation Rate from Vessels	*α*_*mn*_	0.40 (1/day)	[47]
Tumour Vasculature			
Diffusivity of Blood Vessels	*D*_*m*_	1.75e-4 (mm^2^/day)	[47]
Vessel Production Rate	*α*_*nm*_	4.38e-8 (1/day)	[40]
Vessel Chemotaxis Rate	*β*_*nm*_	4.40e-10 (mm^2^/day)	Estimate^1^
Vasculature Scale	*m*_*lim*_	$\sqrt{2}$	[47]
Vessel Efficiency Factor	*γ*	-0.35 (1/day)	[47]
Vessel Efficiency Factor	*δ*	1.05 (1/day)	[47]
Vessel Efficiency Factor	*ϵ*	-0.70 (1/day)	[47]
Oxygen			
Diffusivity of Oxygen	*D*_*k*_	1.05e-3 (mm^2^/day)	[47]
Oxygen Supply Rate	*r*_*k*_	0.280 (1/day)	[47]
Max Oxygen Consumption Rate	*q*_*k*_	1.75e-8 (mmHg/cell/day)	Estimate^2^
Half-Max $P_{O_{2}}$ Consumption	*k*_*c*_	10.0 (mmHg)	[27]
Oxygen Decay Rate	*η*	7.14e-2 (1/day)	[47]
Anti Angiogenesis Drugs			
Diffusivity of Anti Angiogenic	*D*_*A*_	3.46 (mm^2^/day)	[40]
Transvascular Diffusivity	λ_*A*_	0.35 (1/day)	[40]
Anti Angiogenic Decay Rate	*k*_*A*_	0.14 (1/day)	[40]
Hydraulic Conductivity	$\lambda_{l}^{tumour}$	0 (1/mmHg/day)	[40]
Hydraulic Conductivity	$\lambda_{l}^{normal}$	57.54 (1/mmHg/day)	[40]
Hypoxia-Activated Prodrugs			
Diffusivity of Inactive HAPs	*D*_*c*_	2.16e-2 (mm^2^/day)	[27]
Diffusivity of Active HAPs	*D*_*a*_	2.16e-2 (mm^2^/day)	[27]
Inactive HAP Supply Rate	*r*_*c*_	0.07 ([HAP]/day)	[27]
Max HAP Activation Rate	*q*_*c*_	726 (1/day)	[27]
$P_{O_{2}}$ for Half-Max Activation	*k*_*a*_	5.00 (mmHg)	[27]
Inactive HAP Decay Rate	λ_*c*_	2.30e-2 (1/day)	[27]
Active HAP Decay Rate	λ_*a*_	2.30e-2 (1/day)	[27]
Death Rate Due to HAP	*δ*_*a*_	10.0 (1/[HAP]/day)	[27]
Radiation			
Max Oxygen Enhancement	*α*_1_	1	[50]
Min Oxygen Enhancement	*α*_2_	1/3	[50]
$P_{O_{2}}$ for Half-Max Radiation	*k*_*s*_	3.00 (mmHg)	[50]
LQ Model Linear Constant	*α*	0.10 (1/Gy)	[50]
LQ Model Quadratic Constant	*β*	0.03 (1/Gy^2^)	[50]
Radiation Dose Rate	*D*	8 (Gy/day)	[20]

^1^ The term involving *β*_*nm*_ originally comes from [40] who used a finite difference scheme to solve their model. The difference in spatial discretization necessitates an alteration of the value of *β*_*nm*_. Its new value is estimated to qualitatively match the vasculature obtained in [40].

^2^ *q*_*k*_ was changed from the value in [47] to increase the effect of combretastatin and make the differentiation between cases of AAs more pronounced. More details on the estimation for both parameters can be found in the appendix.

**Table 2:** Model parameters.

Parameter	Symbol	Value (Unit)	Reference
Nanocell Supply Rate	*R*_*N*_	0.28 (1/day)	[39]
Nanocell Decay Rate	λ_*N*_	0.18 (1/day)	Estimate^1^
Anti Angiogenic Release Rate	*P*_*A*_	0.1	[39]
HAP Release Rate	*P*_*c*_	0.3	[39]
Anti Angiogenic Half-Life	*t*_*A*_	0.83 (days)	[39]
HAP Half-Life	*t*_*C*_	0.12 (days)	[27]

^1^ The nanocell decay rate is estimated by matching initial concentrations of combretastatin from the separate and nanocell cases.

### Separate administration model

$$\begin{array}{c} \frac{\partial n(\vec{x},t)}{\partial t}=D_{n}\nabla^{2}n+rn(1-\frac{n}{n_{lim}})+\alpha_{mn}mn-\delta_{a}an-nR\left(t\right) \end{array}$$ (1)

Eq (1) describes the tumour cell density, $n(\vec{x},t)$, at position $\vec{x}$ and time *t*, including a Laplacian diffusion term and logistic growth. These mathematical forms for cell growth and diffusion have been widely used in mathematical modelling of tumours [23, 24, 27, 39, 40, 47, 51–53]. To incorporate the increased growth rate of those cells nearby to higher tumour vessel density, $m(\vec{x},t)$, the coupling term *α*_*mn*_ *mn* is included which allows for the cell density to exceed the carrying capacity imposed by logistic growth. This term is phenomenological and has similarly been used by previous studies ([40] for example). The final two terms of Eq (1) allow for the killing effect of activated HAPs, $a(\vec{x},t)$, and radiation therapy administered on the schedule *R*(*t*).

$$\begin{array}{c} \frac{\partial m(\vec{x},t)}{\partial t}=D_{m}\nabla^{2}m+m\left(\gamma +\delta m+\epsilon m^{2}\right)+\alpha_{nm}nm-\beta_{nm}\nabla \cdot (m\nabla n)-\sigma mA \end{array}$$ (2)

In order to model tumour vasculature, a course-grained model is used to develop the functionally deficient vessels. In Eq (2), $m(\vec{x},t)$ represents the blood vessel density which we assume to evolve in the same way as outlined in previous modelling studies [39, 40, 47]. The term *m*(*γ* + *δm* + *ϵm*^2^) allows for three fixed points under appropriate conditions on the constants (*γ* < 0, *δ* > 0, *ϵ* < 0). For the specific case of *δ* = −3*γ* and *ϵ* = 2*γ*, there are two stable fixed points at *m* = 0 and *m* = 1 and an unstable fixed point at *m* = 1/2. the case *m* = 1 is considered to be vascular and *m* = 0 to be nonvascular. In the absence of tumour cells, the vasculature will simply develop into a web of *m* = 0, 1 with steep drop-offs in the transitions. To model the overvascularization induced by the presence of tumour cells, the terms *α*_*nm*_*nm* and −*β*_*nm*_∇ ⋅ (*m*∇*n*) are included. The first of these models the recruitment of pro-angiogenic factors by tumour cells and the second models the chemotaxis of blood vessels toward the tumour core. Note that these terms indirectly allow for cytotoxic therapies to affect the tumour vasculature through the reduction of pro-angiogenic factors typically up-regulated by tumour cells (as done by others [40, 47]). Importantly, these terms also allow for the over-vascularization (*m* > 1) typical of tumours to occur. The final term of Eq (2) allows for the destruction of vessels through the application of AAs, $A(\vec{x},t)$.

$$\begin{array}{c} 0=D_{k}\nabla^{2}k+r_{k}me^{-{\left(\frac{m}{m_{lim}}\right)}^{2}}-\frac{q_{k}k^{2}}{k_{c}^{2}+k^{2}}n-\eta k \end{array}$$ (3)

**Fig 2:**
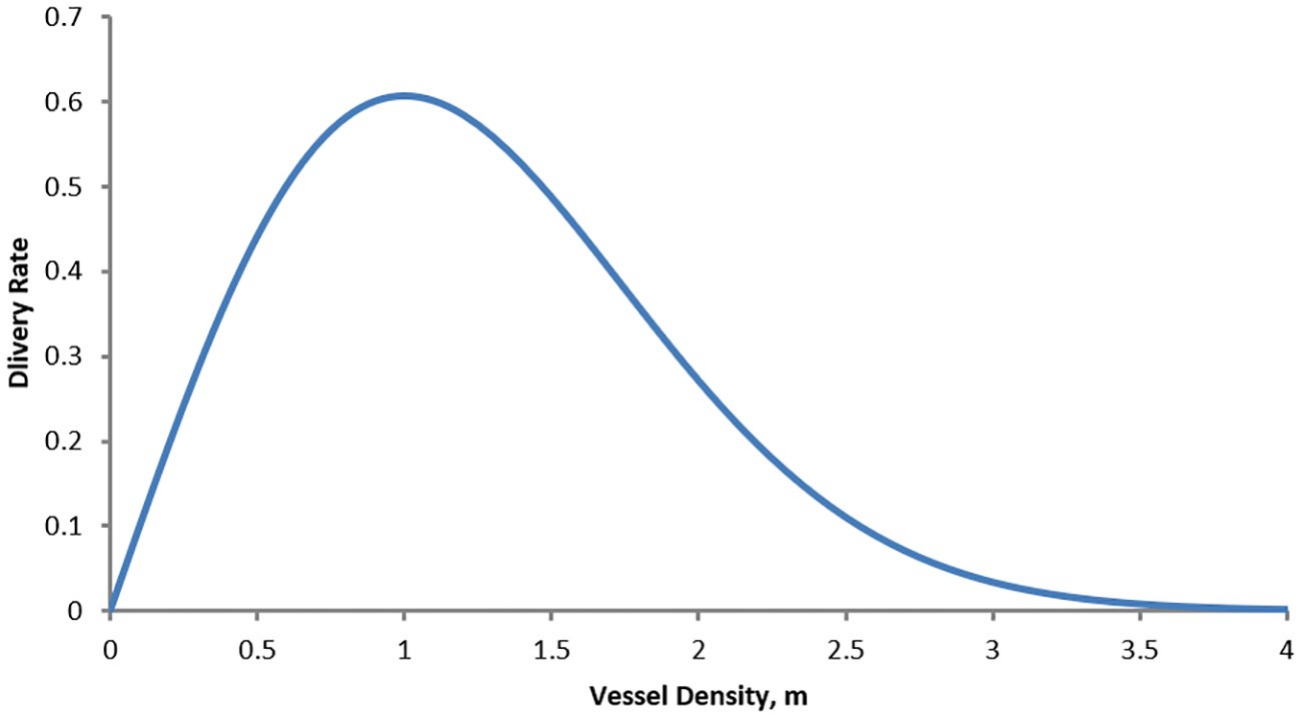
Delivery rate from vessels as a function of the average vessel density. Curve is described by the function $me^{-{\left(\frac{m}{m_{lim}}\right)}^{2}}$. Notice that the optimal vessel delivery occurs at *m* = 1. As *m* becomes greater than 1, the delivery rate decreases, allowing for the impaired delivery due to overvascularization.

Eq (3) describes the partial pressure due to oxygen in the tumour area. The distribution of oxygen is assumed to be in quasi-steady state with respect to the computational time-step length. Importantly, this does not mean that the distribution remains constant throughout the simulation, but rather that it will reach its equilibrium state within the length of a single computational step (which is described below). Eq (3) uses a combination of mathematical terms from Kohandel *et al* 2007 [47], Powathil *et al* 2012 [50], and Meaney *et al* 2019 [27]. Specifically, it is crucial that our model include the effects of decreased oxygen delivery due to overvascularization as well as increased consumption due to cell proximity. However, since no other model has included both effects simultaneously, we use an amalgamation of terms found in previous models to include both effects. Oxygen is assumed to diffuse and decay exponentially at a rate *η*. It is also assumed that, as in [27], oxygen is consumed by tumour cells at a rate dependent on the local oxygen availability. This rate is dictated by a Hill function, a common form in biological modelling. Notice that this consumption rate approaches *q*_*k*_ (its maximum consumption rate) as oxygen concentration increases and 0 as it decreases. For oxygen delivery from vessels, the form $r_{k}me^{-{\left(\frac{m}{m_{lim}}\right)}^{2}}$ is used which allows for a different delivery efficiency depending on the density of tumour vasculature. This term has been utilized in previous modelling studies for delivery of oxygen as well as drugs to tumour sites [39, 47]. Notice that if $m_{lim}=\sqrt{2}$, this delivery curve attains a maximum at *m* = 1 (also see Fig 2 for this delivery curve). With this form, the delivery of oxygen (and HAPs in Eq (5)) is hindered by the over-vascularization in the tumour. Mathematically, the goal of vessel normalization is to bring the value of *m* toward 1 to improve the delivery of drugs and oxygen as controlled by this term.

$$\begin{array}{c} 0=D_{A}\nabla^{2}A+\lambda_{A}m\left(A_{\nu }\left(t\right)-A\right)-\Gamma_{l}\left(n\right)A-k_{A}A,\;\Gamma_{l}\left(n\right)=\{\begin{array}{c} \lambda_{l}^{tumour},\text{if}\;\text{in}\;\text{tumour}\\ \lambda_{l}^{normal},\text{otherwise} \end{array} \end{array}$$ (4)

For combretastain (Eq (4)), the equation outlined by Yonucu *et al* 2017 [40] is adopted which describes the concentration of AAs throughout the tumour area. As with oxygen, combretastatin concentration is assumed to be in quasi-steady state with respect to the computational time step. Combretastatin diffuses and decays exponentially at a rate *k*_*A*_. *A*_*ν*_(*t*) is the combretastatin concentration in plasma which is manually selected according to the appropriate treatment schedule. Diffusion from the vessels occurs with a transvascular diffusivity λ_*A*_. Drainage of combretastatin to the lymph vessels is included such that drainage is hindered in the tumour. This is done with the cell density-dependent parameter Γ_*l*_(*n*) which takes a different value depending on the cell density at that location.

$$\begin{array}{c} 0=D_{c}\nabla^{2}c+r_{c}H\left(t\right)me^{-{\left(\frac{m}{m_{lim}}\right)}^{2}}-\frac{q_{c}k_{a}^{2}}{k_{a}^{2}+k^{2}}c-\lambda_{c}c \end{array}$$ (5)

$$\begin{array}{c} 0=D_{a}\nabla^{2}a+\frac{q_{c}k_{a}^{2}}{k_{a}^{2}+k^{2}}c-\lambda_{a}a \end{array}$$ (6)

Eq (5) models the concentration of inactive TH-302, which is assumed to diffuse and undergo exponential decay. Additionally, TH-302 is transported into the system through the imperfect delivery term as in the case of oxygen in Eq (3). The factor *H*(*t*) is the administration schedule for HAP treatment which is manually assigned. The activation of TH-302 to Br-IPM is modelled by the term $-\frac{q_{c}k_{a}^{2}}{k_{a}^{2}+k^{2}}c$ in Eq (5) and its negative in Eq (6).

$$\begin{array}{c} O\left(k\right)=\frac{\alpha_{1}k+\alpha_{2}k_{s}}{k+k_{s}},\;R\left(t\right)=\{\begin{array}{cc} {}[\alpha +\beta O(k)D]O\left(k\right)D, & \text{during}\;\text{radiation}\\ 0, & \text{otherwise} \end{array} \end{array}$$ (7)

To model the effect of radiation therapy on the tumour, an oxygen enhancement ratio (OER) is employed as done by others ([23, 27]). This models the phenomenon of radiation therapy having a larger effect on well-oxygenated cells than on hypoxic cells because of the creation of oxygen free-radicals which cause DNA damage. As can be seen in the first part of Eq (7), as oxygen concentration increases, the OER approaches the value of *α*_1_, and as oxygen concentration decreases, the OER approaches *α*_2_ (with *α*_1_ > *α*_2_). Accordingly, radiation effect is given by the well-known linear-quadratic (LQ) model for radiation efficacy as in the second part of Eq (7) with the radiobiological parameters *α* and *β* [54].

### Nanocell administration model

The separate administration model can be extended to include nanocell delivery. The equations for cells, vasculature, and oxygen remain unchanged, but the equations for Combretastatin and TH-302 require slight alterations. First, an equation for nanocell concentration, $N(\vec{x},t)$, is added which involves only transport into the system and exponential decay. The production term is given by the same imperfect delivery form as utilized previously for oxygen and drug transport. *N*_*ν*_(*t*) is manually assigned and controls the schedule of administration. Since TH-302 and combretastatin are released from nanocells, the production terms from Eqs (4) and (5) are altered such that the molecules emerge from nanocells instead of vessels. Additionally, *S*_*A*_(*t*) and *S*_*C*_(*t*) are introduced which are the release profiles of the combretastatin and TH-302 from nanocells, respectively. The forms of the release profiles are taken from Kohandel *et al* 2011 [39] who chose them based on the experimental results by Sengupta *et al* 2005 [42]. Sengupta *et al* found that the release of doxorubicin was delayed relative to the release of the combrestatin. The forms of the release profiles are given below where *N*_*C*_ and *N*_*A*_ are the normalization constants and $t_{0}^{\left(A\right)}$ and $t_{0}^{\left(C\right)}$ are the start time of the release profiles relative to the nanocells which are selected based on the administration schedule. These normalization constants are included to ensure a fair comparison between the administration types and more details on their derivation can be found in the appendix. The equations remain unchanged otherwise. They can be seen below in Eqs (8)–(10).

$$\begin{array}{c} 0=R_{N}N_{\nu }\left(t\right)me^{-{\left(\frac{m}{m_{lim}}\right)}^{2}}-\lambda_{N}N \end{array}$$ (8)

$$\begin{array}{c} 0=D_{A}\nabla^{2}A-\lambda_{A}mA-\Gamma_{l}\left(n\right)A-k_{A}A+R_{A}NS_{A}\left(t\right),\;S_{A}\left(t\right)=N_{A}{\left(t-t_{0}^{\left(A\right)}\right)}^{P_{A}}e^{-\left(t-t_{0}^{\left(A\right)}\right)/t_{A}} \end{array}$$ (9)

$$\begin{array}{c} 0=D_{c}\nabla^{2}c+R_{c}NS_{C}\left(t\right)-\frac{q_{c}k_{a}^{2}}{k_{a}^{2}+k^{2}}c-\lambda_{c}c,\;S_{C}\left(t\right)=N_{C}{\left(t-t_{0}^{\left(C\right)}\right)}^{P_{C}}e^{-\left(t-t_{0}^{\left(C\right)}\right)/t_{C}} \end{array}$$ (10)

### Numerical implementation

**Fig 3:**
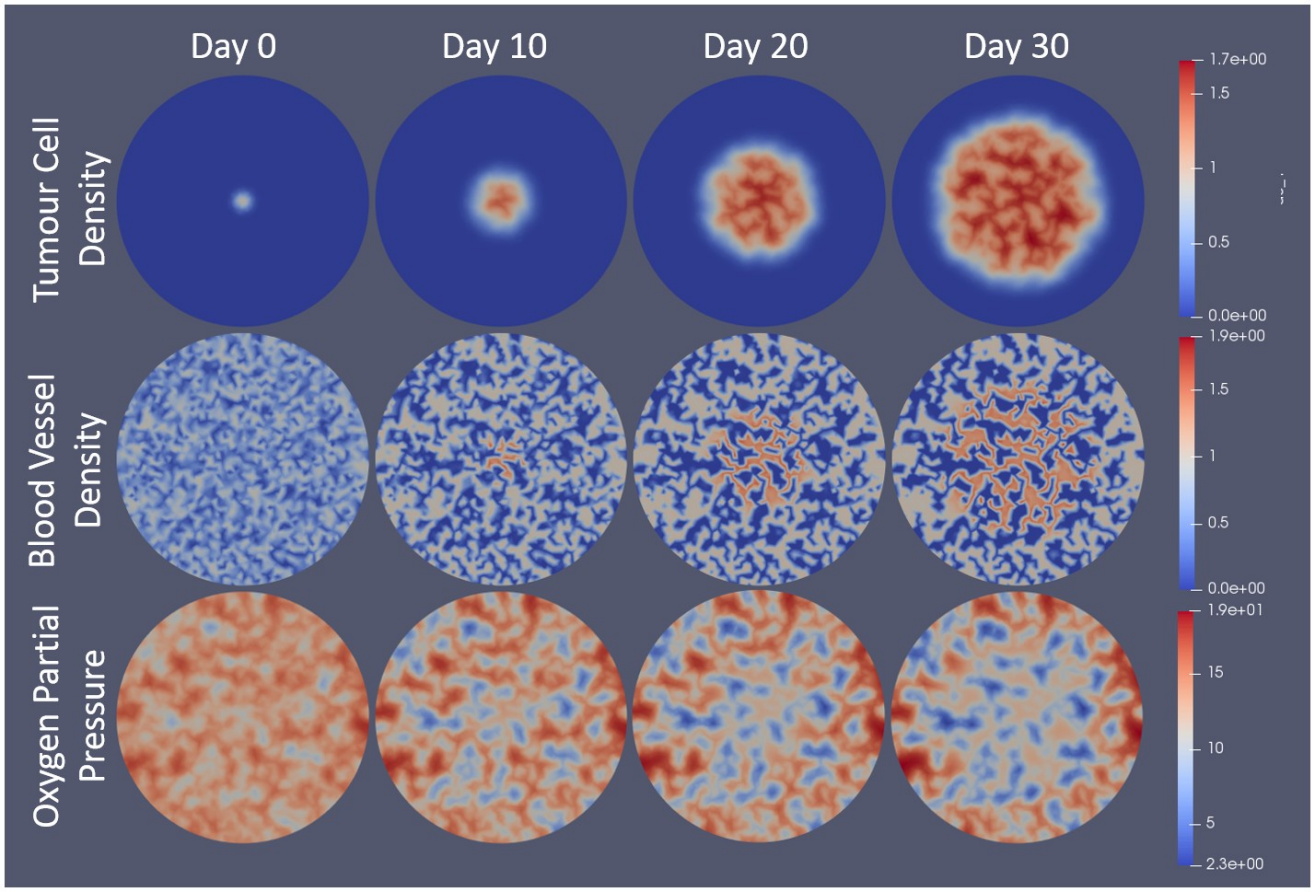
Visualization of model results for tumour cell density, blood vessel density, and oxygen partial pressure over a 30 day span with no treatment. Model is solved using the FEniCS Project Finite Element Solver [55, 56] and is visualized in Paraview [57]. Tumour cells begin as a normalized Gaussian distribution with a standard deviation of approximately 0.63mm. Vessels start randomly distributed between 0 and 1 over the domain. Oxygen is solved in quasi-steady state with respect to the time step length. Day 0 corresponds to the solution of the system after the first time step. Notice that vessels grow toward islands or tubes of density 0 and 1 in the absence of tumour cells, but will become overvascularized (*m* > 1) in the presence of tumour cells. Also notice that the oxygen concentration decreases in tumour area due to the overvascularization. Scales on the right correspond to the nondimensional units.

The model was computationally implemented using a second-order accurate continuous Galerkin finite element method with Euler time-stepping solved on a circular domain. The FEniCS Project Finite Element PDE Solver [55, 56] was used to solve the system using a Neumann (no flux) boundary condition on the circular domain boundary. The system was nondimensionalized prior to solving (details in appendix). For initial conditions, the tumour cell density begins with a small Gaussian distribution at the centre with a standard deviation of approximately 0.63mm (2 nondimensional units). Vessels begin randomly distributed over the domain between 0 and 1. From there, they evolve into the normal vascular structure. The rest of the model equations are solved in quasi-steady state and therefore do not require initial conditions. The administration schedules of the drugs and nanocells are added computationally with temporal step functions. Γ_*l*_ in the equations involving combretastatin is computationally applied using a ‘smoothed’ step-function to maintain continuity so as to avoid computational errors. The drug concentrations in the plasma are assumed to decay exponentially. The schedules used will be pointed out as appropriate in the results section below. The normalization constant used in the release profiles from nanocells is included such that the total drug administered to the patient is equal so that a fair comparison between the two methods can be made. Importantly, this does not mean that the total drug concentration is the same. In particular, in the cases involving vascular normalization, the point of using AAs is to improve delivery and therefore have more drugs extravasated to the tumour area. Rather, the normalization of the drug schedules ensures that the same amount of drug would enter the system if the vessel distributions were identical. For all simulations, the same initial conditions were used (see Fig 3). A time step length of 1 hour was used for most simulations and 10 minutes for those involving radiation therapy. The unstructured spatial grid was generated such that there were 32 elements along the radius of the circular domain which had a radius of 10mm. The run time of the simulations was on the order of a few hours, depending on the specific simulation case.

## Results

### Preliminary results

**Fig 4:**
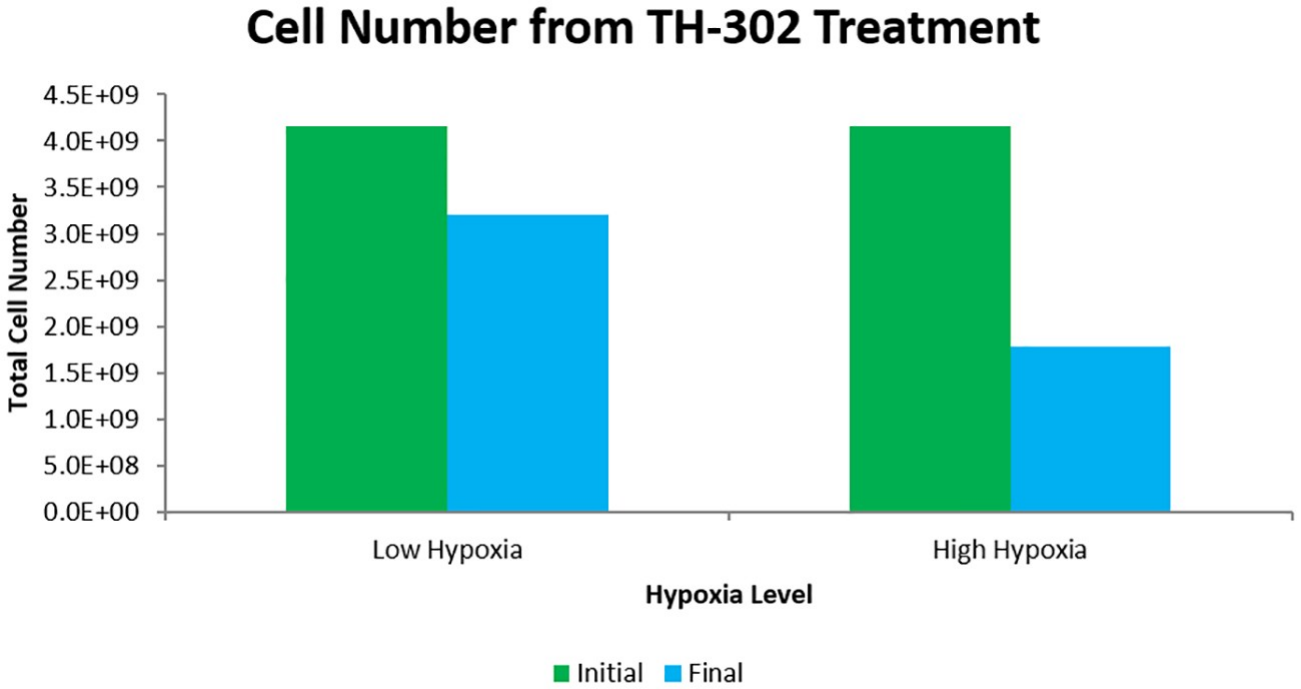
Comparison of TH-302 effectiveness for tumours of different hypoxic levels. The tumour is allowed to grow for 15 days before TH-302 treatment. TH-302 is given every other day starting on the 15th day and the plasma concentration is assumed to have a half life of 3 hours. The high hypoxic case is from the parameters listed in Table 1 while the low hypoxia case assumes a halved oxygen supply rate (*r*_*k*_). Initial cell number is taken from the time step immediately before treatment and final cell number is taken from the time step immediately following treatment. TH-302 treatment kills only 23% of tumour cells in the low hypoxic case while it kills 57% of cells in the high hypoxic case.

To test the model, previously obtained results from experimental and mathematical studies were reproduced. The simplest of these was that TH-302, singly administered, is more effective in a tumour with a higher level of hypoxia. To show this, two tumours were simulated from identical initial conditions for 15 days with different oxygen supply rates, *r*_*k*_. One tumour had the supply rate as reported in Table 1, the other had a supply rate half of that. To these tumours, 5 doses of TH-302 were given in two-day increments starting on the 15th day (days 15, 17, 19, 21, and 23) where the plasma concentration of TH-302 is assumed to halve every 3 hours (as in [27]). The results show that treatment on the high-hypoxia tumour kills 57% of the tumour cells while treatment on the low-hypoxia tumour kills only 23% of the cells. These results can be seen in Fig 4. This matches with the qualitative results as obtained by Meaney *et al* 2019 [27] who performed the same numerical experiment with a different mathematical model. Importantly, the mathematical model used in that study did not include an equation for vessel distribution, but rather used a constant vasculature obtained from imaging. Also, that study used a finite difference scheme to solve the model whereas a finite element method was used here. Though this result is unsurprising based on our understanding of the action of HAPs, we believe that it is important to include as it not only acts as a check on the qualitative predictions of our model, but also stresses the importance of pre-treatment knowledge of the hypoxia level of the tumour when predicting the effectiveness of HAPs.

**Fig 5:**
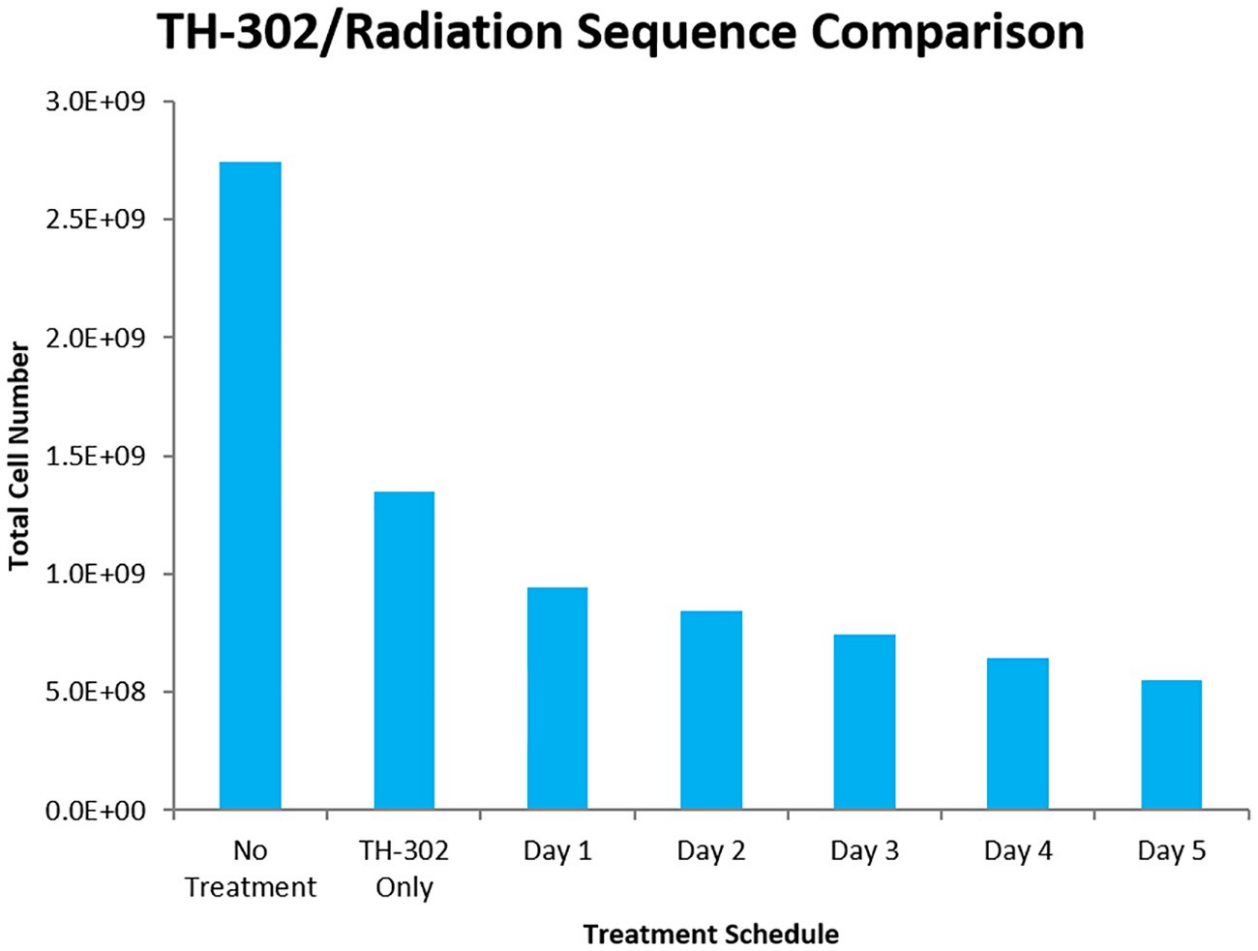
Efficacy of TH-302/radiotherapy combinations based on administration sequence. The tumour is allowed to grow for 15 days with TH-302 treatment starting on the 15th day. TH-302 is then given every day for 5 straight days with radiation given 6 hours after TH-302 on one of the days. Resulting cell number is calculated at the end of the 5th day, after all treatment has ceased. Notice that the later radiotherapy is administered, the more tumour cells are killed.

Another preliminary result examined was the combination of TH-302 with radiation therapy. Specifically, Peters *et al* [20] administered TH-302 with radiotherapy to rabdomyosarcoma R1 and H460 NSCLC and showed that the combination was an improvement over either therapy administered singly. Furthermore, they showed that a single 8 Gy dose of radiation was most effective when given after administration of TH-302, rather than prior to or simultaneously with TH-302. They hypothesized that HAP treatment reduced the hypoxic fraction of cells, thus leading to more oxygen free-radicals which improved the radiation efficacy. Nytko *et al* [19] determined the same qualitative result by showing that neoadjuvant (HAP-first) therapy was optimal when combined with radiotherapy. Meaney *et al* [27] were able to produce the same qualitative results using mathematical modelling. They showed that for TH-302 given once per day for five straight days, the optimal timing for radiotherapy was on the 5th day after the final dose of HAPs. This would align with the hypothesis suggested by [20] and echoed by [19]. Here, our model was used to reproduce the findings of [27] so as to further verify the action of TH-302 in our system. The treatment schedule used was exactly that as in [27] with TH-302 given on each of 5 straight days with 8 Gy of radiation given on one of the days. This can be seen in Fig 5.

**Fig 6:**
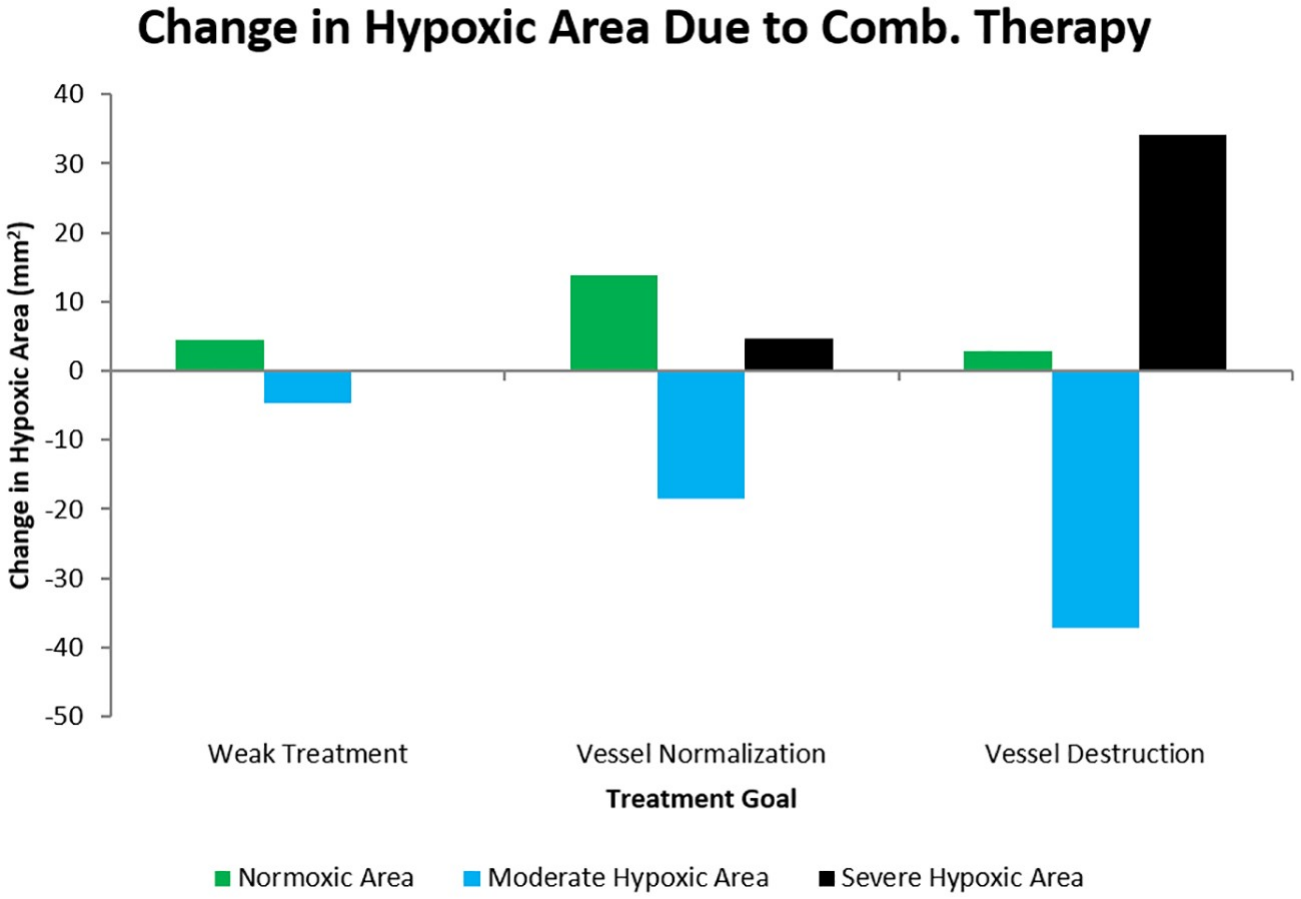
Cases of anti angiogenic therapy effect based on combretastatin strength. Weak treatment corresponds to an anti angiogenic strength parameter of *σ* = 20, vessel normalization to *σ* = 30, and vessel destruction to *σ* = 70. Normoxic area is where [*O*_2_] > 10mmHg, moderate hypoxic where 5 < [*O*_2_] < 10mmHg, and severe hypoxic where [*O*_2_] < 5mmHg. The hypoxic ranges were chosen from the understood ranges of tumour hypoxia ([7, 25] for example). Combretastatin is given every other day starting on the 15th day in decreasing amounts (of magnitudes 1, 0.75, 0.5, 0.25, and 0.1). Notice that there is an optimal middle strength where the increase in normoxic area is maximized.

Next, we used the model to simulate the effect of single administration of combretastatin given in the same schedule as in Yonucu *et al* 2017 [40] who gave 5 daily doses in decreasing amounts (of magnitudes 1, 0.75, 0.5, 0.25, and 0.1) with a half life of 20 hours as used in [27]. From this, the anti angiogenic strength parameter, *σ* was altered to generate different cases of drug effect. The change in hypoxic area was examined in order to observe the phenomenon of application of anti angiogenic agents resulting in an improvement in oxygen distribution. The results are shown in Fig 6 where three distinct cases of effects can be seen. For a weak combretastatin effect, there is a small increase in normoxic area ([*O*_2_] > 10mmHg) and a small decrease in the amount of moderate hypoxic area (5 < [*O*_2_] < 10mmHg) in addition to a very small increase in severe hypoxic area ([*O*_2_] < 5mmHg). The hypoxic ranges were chosen from the accepted ranges in literature ([7, 25] for example). Generally, areas which are severely hypoxic are diffusion-limited and those which are moderately hypoxic are perfusion-limited. As the strength of the combretastatin increases, there is a point where the increase in normoxic area (and corresponding decrease in perfusion limited hypoxia) is optimized. Beyond this point, the normoxic increase declines and the area becomes diffusion limited and severely hypoxic. This shows the ability of the model to produce the different cases of AA effect. Very strong AA strength causes vascular destruction which greatly reduces the oxygen. For intermediate AA strength, optimal oxygen proliferation is achieved through vasculature normalization by pruning $m(\vec{x},t)$ such that it is brought closer to 1.

### Novel results

**Fig 7:**
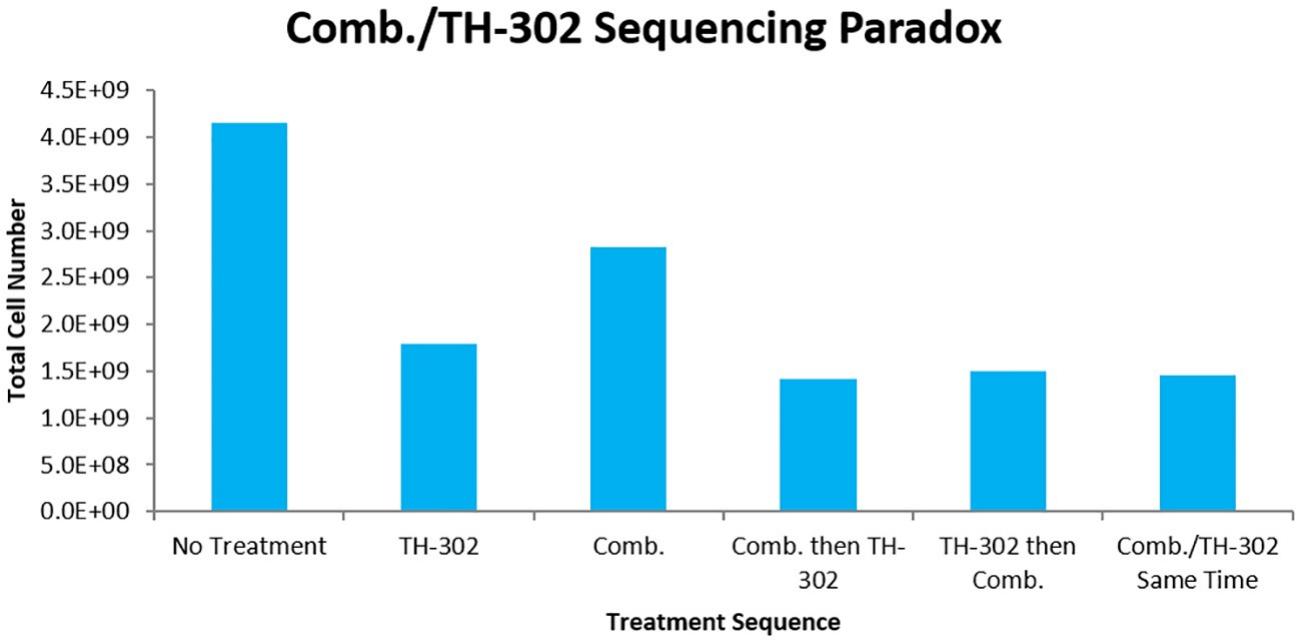
Combretastatin/TH-302 sequencing paradox with vessel normalization. The tumour is allowed to grow for 18 days before the start of treatment. Five doses of drugs are given in 2 day increments, each at the same strength. If both types of drugs are given non-simultaneously, then there is a 6 hour delay between their administrations. The optimal schedule is to give combretastatin 6 hours prior to TH-302, although it is only slightly better than other sequences. Furthermore, the combination therapy fails to show any drug synergy effects.

With the preliminary results, the paradox of AA/HAP sequencing can be examined. The application of a strong dose of AAs will result in a large increase in hypoxic area, but the destruction of tumour vessels will prevent effective delivery of HAPs to the tumour. Accordingly, the normalization case of AA effect is used in the combinations in attempt to produce optimal cell kill. In theory, appropriate administration of combretastatin prior to TH-302 should improve TH-302 delivery (through normalization process), while impairing TH-302 activation to Br-IPM. Conversely, if TH-302 is given first, the delivery from vessels will be sub-optimal. In the simulations, treatment schedules began on the 18th day with doses being given every other day. In the sequencing cases, the drug types were either given at the same time or six hours apart on each of the days. Many different sequencing schedules were simulated, and the 6 hour interval produced the largest sequencing effect. Specifically, leaving a longer time between administration of combretastatin and TH-302 allowed the normalization window to close before TH-302, and a shorter time did not allow for the vasculature to be fully normalized. When simulated, the model shows that the combination improves cell kill, but that this improvement is significantly smaller than the sum of the individual effects of the combretastatin and TH-302. This can be seen in Fig 7. The combretastatin-first treatment gives the highest cell kill, albeit slightly, compared to the other sequences. This improved the cell kill to 66%, which is notably less than the sum of the effect of combretastatin (32%) and TH-302 (57%) individually.

**Fig 8:**
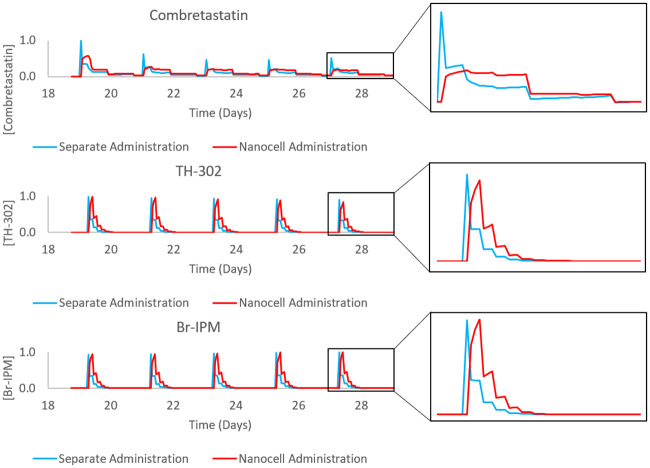
Normalized drug concentrations in the tumour area over the treatment schedule for combretastatin-first treatment administered either separately or through nanocells. The left column of plots is the full treatment schedule used in simulations, the right column is a zoomed-in picture of the final drug administration to better observe the difference in release profiles. Notice the delayed release in the nanocell administration case.

**Fig 9:**
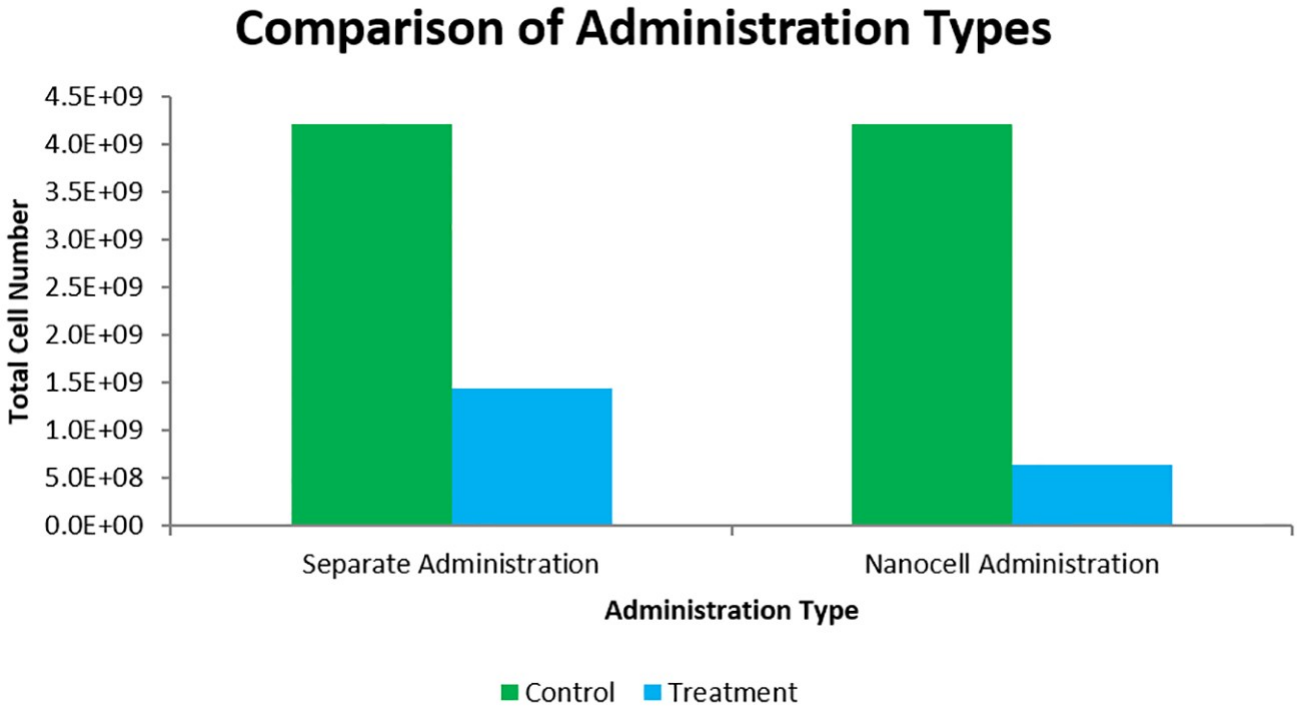
Comparison of optimal separate administration sequence vs. nanocell administration. Treatment schedules are as shown in Fig 7. Notice the improvement in cell kill in the nanocell administration.

To improve the combination, nanocell administration was simulated using the model outlined in Eqs (8)–(10). The schedule used was the same as in the combretastatin-first sequence in Fig 7. Ideally, nanocell administration will result in improved TH-302 delivery while retaining the hypoxia needed for activation to Br-IPM. The resulting drug concentrations from the different administration methods can be seen in Fig 8. Notice that the release profile from nanocells produces a different drug concentration profile than separate administration. Importantly, the concentration of drugs in the plasma remains constant between the methods while the concentration seen by the tumour depends on the perfusion rate from vessels. The plasma concentration is kept constant through normalization factors in Eqs (9) and (10) (details in S1 Text). The difference in cell kill can be seen in Fig 9 where the nanocell delivery improves cell kill from the 66% reported above to 85%. Although the 85% still falls below the sum of the individual effects, a 19% increase in cell kill is a notable improvement.

## Discussion

Our model simulations suggest a novel, advantageous method for administration of HAPs: a combination with AAs through nanocell administration. To our knowledge, no other models—mathematical or experimental—have investigated the combination of HAPs and AAs through nanocell delivery. The results of our model clearly show that the nanocell delivery method results in an increase in the efficacy of TH-302, shown through an increase in the total cell kill of the tumour. The model was also used to reproduce the results of previous experiments, which are summarized and compared in more detail below.

Although HAPs have been investigated for several decades, their mainstream application has become popular in recent years primarily due to advancements in various medical technologies. The most attractive quality of HAPs is their specificity towards neoplastic tissue, leading to less patient side effects and maximal observed drug concentration for the tumour. The motivation for their continued development despite limited clinical success is a function of nonspecificity of other conventional chemotherapeutics. The lack of clinical success has been attributed largely to inefficient patient selection in trials, but other improvements are still necessary [15, 16, 28, 30, 31, 58]. For instance, the hypoxia selectivity for many HAPs is simply too low for effective treatment [15]. Furthermore, *in vivo* evaluations of HAPs have taken place over differing delivery methods including intravenous injection, intraperitoneal injection, and oral administration [30]. A deeper understanding of preferred administration methods is needed for clinical advancement of HAPs. Additionally, as we move further into the era of personalized medicine, knowledge of the key reductases involved in the activation, transportation, *etc* of HAPs will be crucial [16, 28, 30]. In all of these endeavors, mathematical models have and can continue to provide valuable insight [13, 23–27, 59]. Hopefully HAPs can progress through the various stages of clinical development, where they currently reside.

Since HAPs remain largely in testing phases, there are few experimental studies examining their combination with AAs. In Yoon *et al* 2015, [43] combined TH-302 and radiotherapy with the AA DC101 to examine combination effects in xenograft mouse models of sarcoma. Their results showed that the addition of TH-302 to the combination treatment improved the blockage of tumour growth, with tumours remaining dormant for up to 3 months after treatment. The TH-302/DC101 combination was shown to significantly decrease the tumour proliferation from the individual cases. It is unclear whether or not there was any evidence of drug synergy, or whether the effect of combination was simply the sum of the effects of the individual drugs. Furthermore, the effects of treatment sequencing were not examined, with DC101 always being given 2 hours prior to TH-302 or radiation. Vessel normalization for the purpose of enhancing drug delivery was also not discussed, rather DC101 was given mainly for the purpose of oxygenating the tumour and improving radiotherapy. Following this, Yoon *et al* 2016 [44] combined TH-302 with pazopanib in genetically engineered and xenograft mice, showing that the multimodal therapy enhanced the treatment in all cases. Sequencing, nor vessel normalization were discussed here as well. In Liu *et al* 2017, [45] combined TH-302 with the AA sunitinib in either short-term or long-term treatment, with the two being given at the same time. In the long-term treatment, the combination was more effective in reducing tumour volume than either drug individually, but there was no evidence of drug synergy as, over long treatment, each drug resulted in a large drop in tumour volume already. In the short-term case, the effect of the sunitinib individually was negligible, however when combined with TH-302 resulted in a dramatic decrease in tumour volume. Interestingly, the investigators assumed the increased efffect of the TH-302 to be due to an increase in hypoxia from the sunitinib. However, there was no discussion of drug delivery changes due to vessel normalization. The presence of drug synergy in the short-term case and not the long-term case is perhaps suggestive that vessel normalization occurred in such a way that drug delivery was optimized with a minimal reduction in hypoxic area. Finally, Kumar *et al* [46] again combined TH-302 with sunitinib, but no drug synergy was evident. They even speculated that the lack of drug synergy was perhaps due to vessel normalization which reduced the hypoxia such that the TH-302 was less effective.

Vessel normalization techniques are a relatively new concept in oncology, although the theory is well-understood and many experimental studies have examined its effect ([38, 60, 61] for example or [33, 62] for recent reviews). Through these, it has become abundantly clear that normalizing, rather than destroying tumour vasculature is the preferred mode of treatment [62]. To rationalize this phenomenon, it is helpful to understand that tumour oxygenation is one of the best indicators of patient survival, largely through its ability to promote aggression and metastasis, further immunosuppression, and enhance treatment resistance [1, 3, 5, 10, 33, 62]. It is then reasonable that reoxygenating a tumour could improve patient survival. This is often done through VEGF inhibitors—the action of most AAs. The AA examined here, combretastatin, has a different effect which is to suppress the capillary-like tube formation of endothelial cells by attacking vessel walls (although it appears to have some VEGF blocking capabilities as well) [63]. As such, combretastatin was modelled as attacking vessels from within (as was done in [40]).

In this study, we provide a mathematical backbone to a theoretically beneficial treatment method. Specifically, the experiments by Sengupta *et al* [42] clearly demonstrate the superiority of drug delivery to hypoxic regions by nanocells compared to separate administration—however this is shown for conventional chemotherapeutics, not HAPs. Similarly, the various results mentioned above demonstrate that the combination of AA/HAP treatment has potential to be a powerful one, but that more experimental studies are necessary in order to fully understand the mechanisms at play. Furthermore, the little available evidence suggests that drug synergy is possible, but that precise scheduling and dosing is crucial to success. What has not been examined thus far—experimentally or mathematically—is the use of nanocells as a delivery mechanism for HAPs. Our mathematical study attempts to do just this. Our results indicate that nanocell administration may be beneficial in realizing the right dosing and scheduling and be able to increase HAP effectiveness through utilizing the increased delivery and activation.

As with any mathematical model, ours is not perfect, nor do we claim it to be. Specifically, the model can only be used reliably for qualitative predictions. This is largely due to the imprecision of parameter measurements and the variability of parameters between patients. The key parameters used in our system have all been subject to sensitivity analyses within the works from which they come. We have supplemented this with additional analyses related to the conclusion of this work. Specifically, how sensitive are the measures of cell kill and hypoxic area to parameter perturbations, and can these sensitivities affect the overall conclusions? The sensitivity analyses show that the model is robust with respect to reasonable changes in the model parameters (of ±10% to the parameter value—see S1 Text, S1 and S2 Figs for more details). These analyses can be seen in the appendix.

Although the mathematical assumptions made have generally been made by others as well, one could legitimately question their validity. The infinite diffusion speed imposed by Laplacian diffusion is obviously non-physical. Similarly, the immediate, rather than delayed effect of drugs and radiation is not biologically reasonable. The delayed effect could be incorporated using a technique such as delayed differential equations, which would be an interesting inclusion to a followup study. One could also question the validity of using a vessel density field to model vasculature. It should be stressed again that these are mathematical approximations made in order to derive qualitative predictions, not precise measurements used for quantitative analysis. Ideally, a similar analysis can be done using imaged vasculature which can be imported into the model, but this is obviously far more difficult and costly.

### Conclusion

The use of HAPs has increased greatly in recent years due to the advancement of medical technologies and biological understanding. With the slow pace of HAPs in clinical trials, the need has arisen for novel methods of improving HAP efficacy. One potential avenue for improvement is through the use of AAs which could improve drug delivery through normalizing the abnormal and inefficient tumour vasculature. Unfortunately, in this lies an apparent paradox of increased HAP delivery leading to decreased HAP activation. Although there may exist an optimal middle ground between the two extremes, this work investigates drug nanocells, an alternative approach which has the potential to achieve both increased delivery and activation. This is done through a mathematical analysis, building off many previous modelling studies and using experimental results to inform model approximations and parameters. The model is able to reproduce known results of HAPs, AAs, and radiotherapy, which help to inform the model. The major novel result of our model is a predicted increase in AA/HAP combination efficacy through the use of drug nanocells from the separately applied cases, regardless of sequencing. Hopefully, models such as this can inspire further exploration into the combinations of HAPs with AAs in order to accelerate HAPs through clinical trials. One such way to do this could be through the use of drug nanocells, as is outlined here.

## Supporting information

S1 Fig Relative sensitivities of model parameters for the no treatment case. The relative change in total tumour cell number and total hypoxic area is shown for a tumour after 15 days of growth. Oxygen parameters are only included in the hypoxic area figure as they do not impact the total cell number.
(TIF)

S2 Fig Examination of the effect of perturbations of the model’s most sensitive parameters on the overall conclusion of the model. As can be seen, the nanocell administration is superior to the separate administration in all cases. Each group of bars represents the changes in a single parameter value where the blue bar represents the nanocell case with a 10% increase in the parameter value, the red is separate administration with a 10% increase, green is nanocell with a 10% decrease, and purple is the separate with a 10% decrease. The remaining cell number is normalized using the separate administration case with no parameter changes.
(TIF)

S1 Text (PDF)
