# Supplementary figures and images for: In silico analysis of hypoxia activated prodrugs in combination with anti angiogenic therapy through nanocell delivery
Source: PLoS Comput Biol. 2020 May 28;16(5):e1007926. doi: 10.1371/journal.pcbi.1007926 (PMC7282674; doi:10.1371/journal.pcbi.1007926)

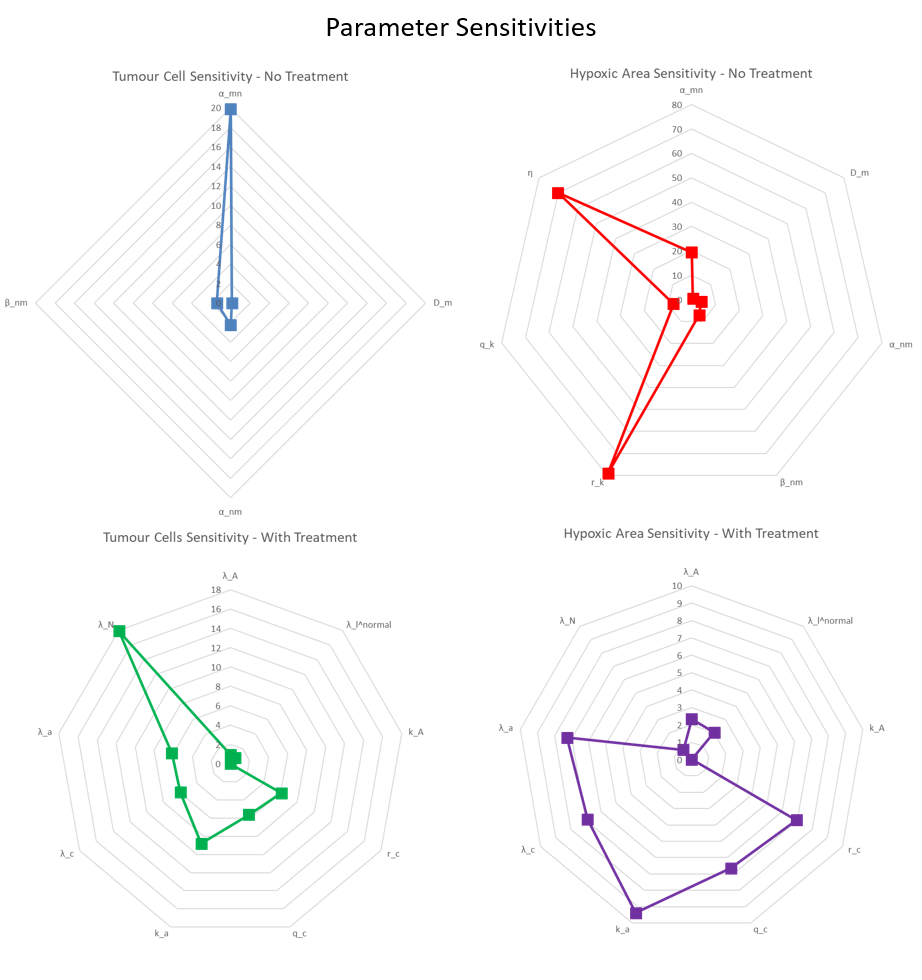

Supplement: S1 Fig — The relative change in total tumour cell number and total hypoxic area is shown for a tumour after 15 days of growth. Oxygen parameters are only included in the hypoxic area figure as they do not impact the total cell number. (TIF) [file pcbi.1007926.s001.tif]

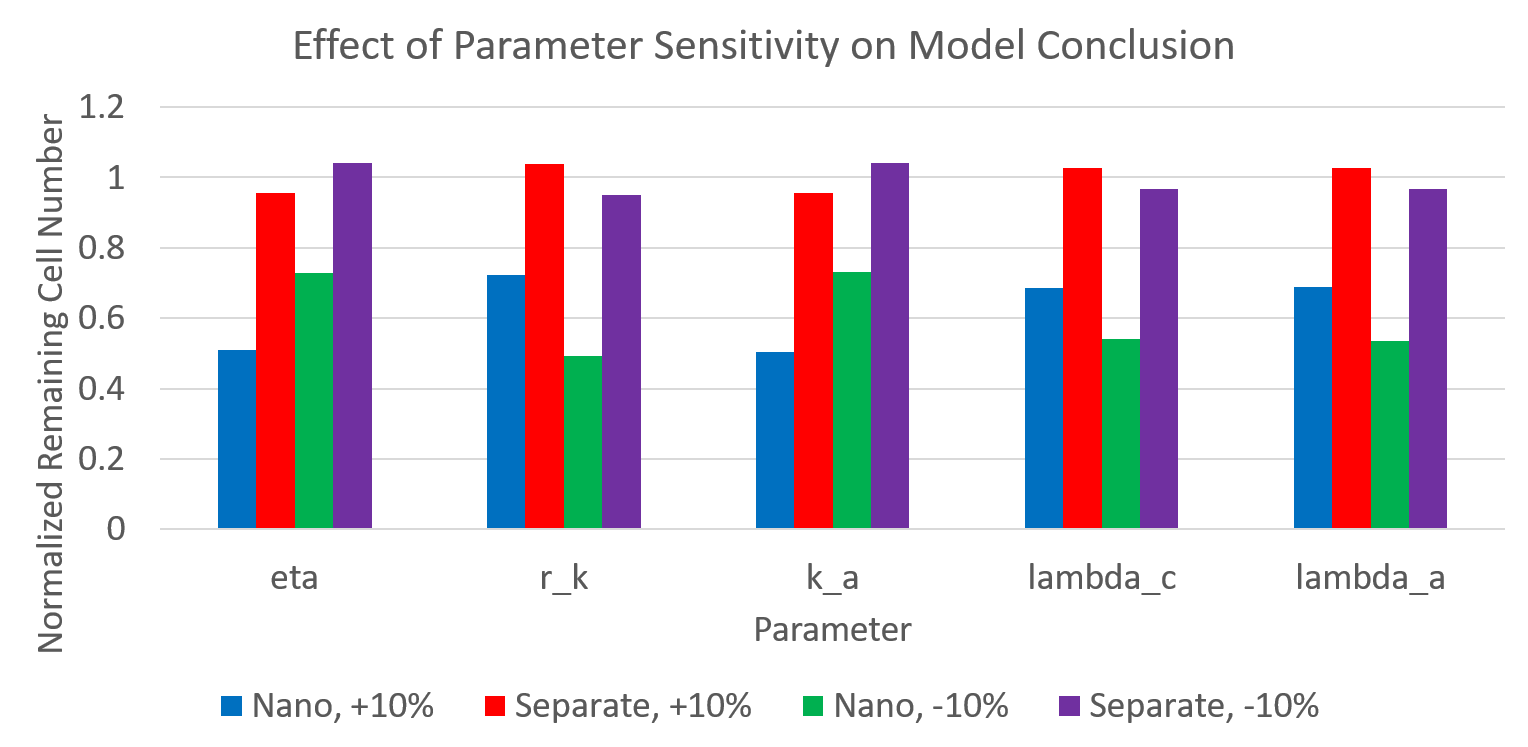

Supplement: S2 Fig — As can be seen, the nanocell administration is superior to the separate administration in all cases. Each group of bars represents the changes in a single parameter value where the blue bar represents the nanocell case with a 10% increase in the parameter value, the red is separate administration with a 10% increase, green is nanocell with a 10% decrease, and purple is the separate with a 10% decrease. The remaining cell number is normalized using the separate administration case with no parameter changes. (TIF) [file pcbi.1007926.s002.tif]
