# Supplementary material for: In silico analysis of hypoxia activated prodrugs in combination with anti angiogenic therapy through nanocell delivery
Source: PLoS Comput Biol. 2020 May 28;16(5):e1007926. doi: 10.1371/journal.pcbi.1007926 (PMC7282674; doi:10.1371/journal.pcbi.1007926)
Supplement: S1 Text — (PDF) [file pcbi.1007926.s003.pdf]

Supplementary Material

Derivation of Release Profile Normalization Constant

To ensure a fair comparison between the administration types, the plasma concentration of the drugs should be the same. To do this, the release profiles are integrated. For the separate administration:

$$\int_{t_0}^{\infty} e^{-\frac{(t-t_0)}{t_i}} dt = t_i, \tag{1}$$

where t_i is the half life of the drug. Then for the nanocell administration:

$$\int_{t_0}^{\infty} (t-t_0)^{P_i} e^{-\frac{(t-t_0)}{t_i}} dt = t_i^{1+P_i} \Gamma(1+P_i). \tag{2}$$

So, equating these with a normalization constant implies that the normalization constant for each drug must be:

$$N_i = \frac{1}{t_i^{P_i} \Gamma(1+P_i)}. \tag{3}$$

This is the form of the normalization constant used to implement the drug schedules.

System Parameters and Sensitivity Analysis

The tumour growth/treatment system is nondimensionalized using the transformations

$$x = \tilde{x}\sqrt{\frac{D_n}{r}}, \quad t = \frac{\tilde{t}}{r}, \quad n = \tilde{n} n_{lim}, \quad k = \tilde{k} k_c. \tag{4}$$

This leads to the nondimensionalized system

$$\frac{\partial n(\vec{x},t)}{\partial t} = \nabla^2 n + n\,(1-n) + \alpha_{mn} m n - \delta_a a n - n R(t) \tag{5}$$

$$\frac{\partial m(\vec{x},t)}{\partial t} = D_m \nabla^2 m + m(\gamma + \delta m + \epsilon m^2) + \alpha_{nm} n m - \beta_{nm} \nabla \cdot (m \nabla n) - \sigma m A \tag{6}$$

$$0 = \nabla^2 k + r_k m e^{-\left(\frac{m}{m_{lim}}\right)^2} - \frac{q_k k^2}{1+k^2} n - \eta k \tag{7}$$

$$0 = \nabla^2 A + \lambda_A m (A_\nu(t) - A) - \Gamma_l(n) A - k_A A \tag{8}$$

$$0 = \nabla^2 c + r_c H(t) m e^{-\left(\frac{m}{m_{lim}}\right)^2} - \frac{q_c k_a^2}{k_a^2 + k^2} c - \lambda_c c \tag{9}$$

$$0 = \nabla^2 a + \frac{q_c k_a^2}{k_a^2 + k^2} c - \lambda_a a \tag{10}$$

$$0 = N_\nu(t) m e^{-\left(\frac{m}{m_{lim}}\right)^2} - \lambda_N N \tag{11}$$

where the parameters are grouped into nondimensionalized reduced parameters and all tildes have been dropped for simplicity. The system is implemented computationally using this nondimensionalized version. A sensitivity analysis is performed on the relevant nondimensionalized parameters appearing in equations (5)-(11) in S1 Fig and S2 Fig. The sensitivity analysis is performed by increasing and decreasing each parameter value by 10%, then calculating the resulting range in tumour cell number and hypoxic area. The sensitivity as shown in S1 Fig is the range from parameter perturbation divided by the unperturbed result (relative sensitivity). Notice that for total tumour cell number without treatment, the model is most sensitive to changes in the proliferation rate from vessels, α_{mn}, with a 20% total change in parameter value leading to an approximate 20% total change in cell number. Also notice that the oxygen parameters are not included in the plot for tumour cells as oxygen availability does not directly affect the cell number in our model: rather, the growth of the tumour is phenomenologically impacted by the tumour vasculature. For hypoxic area, notice that the oxygen supply rate, r_k, and the oxygen decay rate, η, have the largest impact with a 20% change in parameter values leading to an approximate 79% and 70% change in hypoxic area. For parameters related to treatment, the model is generally insensitive to parameter changes, with all sensitivities remaining under 10%, except for the nanocell decay rate, λ_N. However, a change in λ_N simply corresponds to a change in the total amount of drug seen by the tumour, since when nondimensionalized, scaling λ_N is equivalent to scaling R_N. Therefore it is a not a fair comparison with the separate administration case. Furthermore, the goal of a sensitivity analysis is to determine whether perturbations of the model parameters would significantly change the conclusions of the model. To assess this, we further examined those parameters that showed high sensitivity by running additional simulations to see whether the nanocell administration remained superior to the separate administration. As can be seen in S2 Fig, the superiority of nanocells is maintained for all perturbations of the most sensitive parameters. This analysis could be done for all parameters, but would require significant computation. It is similarly unlikely to result in a change of the conclusion since the model is less sensitive to the other parameters as can be seen in Fig. S1 Fig. We therefore conclude that the model is robust with respect to reasonable changes in the parameter values.

S1 Fig. Relative sensitivities of model parameters for the no treatment case. The relative change in total tumour cell number and total hypoxic area is shown for a tumour after 15 days of growth. Oxygen parameters are only included in the hypoxic area figure as they do not impact the total cell number.

Two parameters in our model were estimated to better match with previous studies and analyze the results. The vessel chemotaxis rate, β_{nm}, is estimated to qualitatively match the vasculature obtained in [1]. If the value for β_{nm} from [1] is used in our model, the vessel density does not reach the same maximum and the qualitative vessel shape dramatically changes from that observed in [1]. We manually altered β_{nm} in order to match the observations in [1]. For q_k, the value used in [2] caused a very low amount of hypoxic area to be observed in our simulations, making the effect of AAs difficult to differentiate. To help identify the differences, we increased the value of q_k. Importantly, the model is insensitive to changes in both of these parameters, as can be seen in S1 Fig.

S2 Fig. Examination of the effect of perturbations of the model's most sensitive parameters on the overall conclusion of the model. As can be seen, the nanocell administration is superior to the separate administration in all cases. Each group of bars represents the changes in a single parameter value where the blue bar represents the nanocell case with a 10% increase in the parameter value, the red is separate administration with a 10% increase, green is nanocell with a 10% decrease, and purple is the separate with a 10% decrease. The remaining cell number is normalized using the separate administration case with no parameter changes.

References

[1] Yonucu S, Defne Y, Phipps C, Unlu MB, Kohandel M. Quantifying the effects of antiangiogenic and chemotherapy drug combinations on drug delivery and treatment efficacy. PLoS Computational Biology. 2017; p. 1–17.

[2] Kohandel M, Kardar M, Milosevic M, Sivaloganathan S. Dynamics of tumor growth and combination of anti-angiogenic and cytotoxic therapies. Physics in Medicine and Biology. 2007;52(13):3665–3677. doi:10.1088/0031-9155/52/13/001.
